# Supplementary material for: Resolving the full spectrum of human genome variation using Linked-Reads
Source: Genome Res. 2019 Apr;29(4):635–45. doi: 10.1101/gr.234443.118 (PMC6442396; doi:10.1101/gr.234443.118)
Supplement: Supplemental Material [file supp_gr.234443.118_Supplemental_longranger-2.2.zip › longranger-2.2.tar.bz2!/lr2.2-public/lib/stan/cmdstan/stan/doc/api/html/print_8hpp.html]

Stan: src/stan/command/print.hpp File Reference


|  |  |
| --- | --- |
|  | Stan  2.5.0  probability, sampling & optimization |


- Main Page
- Related Pages
- Namespaces
- Classes
- Files

- File List
- File Members

All Classes Namespaces Files Functions Variables Typedefs Enumerations Enumerator Friends Macros Pages

- src
- stan
- command

Functions

print.hpp File Reference

`#include <algorithm>`  
`#include <iostream>`  
`#include <iomanip>`  
`#include <ios>`  
`#include <stan/mcmc/chains.hpp>`

Go to the source code of this file.

|  |  |
| --- | --- |
| Functions | |
| void | compute\_width\_and\_precision (double value, int sig\_figs, int &width, int &precision) |
|  | |
| int | compute\_width (double value, int sig\_figs) |
|  | |
| int | compute\_precision (double value, int sig\_figs, bool scientific) |
|  | |
| int | calculate\_column\_width (const Eigen::VectorXd &x, const std::string &name, const int sig\_figs, std::ios\_base::fmtflags &format) |
|  | |
| Eigen::VectorXi | calculate\_column\_widths (const Eigen::MatrixXd &values, const Eigen::Matrix< std::string, Eigen::Dynamic, 1 > &headers, const int sig\_figs, Eigen::Matrix< std::ios\_base::fmtflags, Eigen::Dynamic, 1 > &formats) |
|  | |
| void | print\_usage () |
|  | |
| bool | is\_matrix (const std::string &parameter\_name) |
|  | |
| std::string | base\_param\_name (stan::mcmc::chains<> &chains, const int index) |
|  | |
| std::string | matrix\_index (stan::mcmc::chains<> &chains, const int index) |
|  | |
| std::vector< int > | dimensions (stan::mcmc::chains<> &chains, const int start\_index) |
|  | |
| void | next\_index (std::vector< int > &index, const std::vector< int > &dims) |
|  | |
| int | matrix\_index (std::vector< int > &index, const std::vector< int > &dims) |
|  | |

## Function Documentation

|  |  |  |  |
| --- | --- | --- | --- |
| std::string base\_param\_name | ( | stan::mcmc::chains<> & | *chains*, |
|  |  | const int | *index* |
|  | ) |  |  |

Definition at line 120 of file print.hpp.

|  |  |  |  |
| --- | --- | --- | --- |
| int calculate\_column\_width | ( | const Eigen::VectorXd & | *x*, |
|  |  | const std::string & | *name*, |
|  |  | const int | *sig\_figs*, |
|  |  | std::ios\_base::fmtflags & | *format* |
|  | ) |  |  |

Definition at line 54 of file print.hpp.

|  |  |  |  |
| --- | --- | --- | --- |
| Eigen::VectorXi calculate\_column\_widths | ( | const Eigen::MatrixXd & | *values*, |
|  |  | const Eigen::Matrix< std::string, Eigen::Dynamic, 1 > & | *headers*, |
|  |  | const int | *sig\_figs*, |
|  |  | Eigen::Matrix< std::ios\_base::fmtflags, Eigen::Dynamic, 1 > & | *formats* |
|  | ) |  |  |

Definition at line 87 of file print.hpp.

|  |  |  |  |
| --- | --- | --- | --- |
| int compute\_precision | ( | double | *value*, |
|  |  | int | *sig\_figs*, |
|  |  | bool | *scientific* |
|  | ) |  |  |

Definition at line 40 of file print.hpp.

|  |  |  |  |
| --- | --- | --- | --- |
| int compute\_width | ( | double | *value*, |
|  |  | int | *sig\_figs* |
|  | ) |  |  |

Definition at line 33 of file print.hpp.

|  |  |  |  |
| --- | --- | --- | --- |
| void compute\_width\_and\_precision | ( | double | *value*, |
|  |  | int | *sig\_figs*, |
|  |  | int & | *width*, |
|  |  | int & | *precision* |
|  | ) |  |  |

Definition at line 10 of file print.hpp.

|  |  |  |  |
| --- | --- | --- | --- |
| std::vector<int> dimensions | ( | stan::mcmc::chains<> & | *chains*, |
|  |  | const int | *start\_index* |
|  | ) |  |  |

Definition at line 130 of file print.hpp.

|  |  |  |  |  |  |
| --- | --- | --- | --- | --- | --- |
| bool is\_matrix | ( | const std::string & | *parameter\_name* | ) |  |

Definition at line 116 of file print.hpp.

|  |  |  |  |
| --- | --- | --- | --- |
| std::string matrix\_index | ( | stan::mcmc::chains<> & | *chains*, |
|  |  | const int | *index* |
|  | ) |  |  |

Definition at line 125 of file print.hpp.

|  |  |  |  |
| --- | --- | --- | --- |
| int matrix\_index | ( | std::vector< int > & | *index*, |
|  |  | const std::vector< int > & | *dims* |
|  | ) |  |  |

Definition at line 183 of file print.hpp.

|  |  |  |  |
| --- | --- | --- | --- |
| void next\_index | ( | std::vector< int > & | *index*, |
|  |  | const std::vector< int > & | *dims* |
|  | ) |  |  |

Definition at line 156 of file print.hpp.

|  |  |  |  |  |
| --- | --- | --- | --- | --- |
| void print\_usage | ( |  | ) |  |

Definition at line 100 of file print.hpp.


---

[ Stan Home Page ]

*© 2011–2014,
Stan Development Team.*
